# Targeting Prostate Cancer Stem Cells with Alpha-Particle Therapy

**DOI:** 10.3389/fonc.2016.00273

**Published:** 2017-01-09

**Authors:** Jens Ceder, Jörgen Elgqvist

**Affiliations:** ^1^Department of Translational Medicine, Lund University, Skåne University Hospital, Malmö, Sweden; ^2^Department of Physics, University of Gothenburg, Gothenburg, Sweden; ^3^Department of Medical Physics and Biomedical Engineering, Sahlgrenska University Hospital, Gothenburg, Sweden

**Keywords:** cancer stem cells, alpha particles, prostate cancer, radioimmunotherapy, targeted therapy

## Abstract

Modern molecular and radiopharmaceutical development has brought the promise of tumor-selective delivery of antibody–drug conjugates to tumor cells for the diagnosis and treatment of primary and disseminated tumor disease. The classical mode of discourse regarding targeted therapy has been that the antigen targeted must be highly and homogenously expressed in the tumor cell population, and at the same time exhibit low expression in healthy tissue. However, there is increasing evidence that the reason cancer patients are not cured by current protocols is that there exist subpopulations of cancer cells that are resistant to conventional therapy including radioresistance and that these cells express other target antigens than the bulk of the tumor cells. These types of cells are often referred to as cancer stem cells (CSCs). The CSCs are tumorigenic and have the ability to give rise to all types of cells found in a cancerous disease through the processes of self-renewal and differentiation. If the CSCs are not eradicated, the cancer is likely to recur after therapy. Due to some of the characteristics of alpha particles, such as short path length and high density of energy depositions per distance traveled in tissue, they are especially well suited for use in targeted therapies against microscopic cancerous disease. The characteristics of alpha particles further make it possible to minimize the irradiation of non-targeted surrounding healthy tissue, but most importantly, make it possible to deliver high-absorbed doses locally and therefore eradicating small tumor cell clusters on the submillimeter level, or even single tumor cells. When alpha particles pass through a cell, they cause severe damage to the cell membrane, cytoplasm, and nucleus, including double-strand breaks of DNA that are very difficult to repair for the cell. This means that very few hits to a cell by alpha particles are needed in order to cause cell death, enabling killing of cells, such as CSCs, exhibiting cellular resistance mechanisms to conventional therapy. This paper presents and evaluates the possibility of using alpha-particle emitting radionuclides in the treatment of prostate cancer (PCa) and discusses the parameters that have to be considered as well as pros and cons of targeted alpha-particle therapy in the treatment of PCa. By targeting and eradicating the CSCs responsible of tumor recurrence in patients who no longer respond to conventional therapies, including androgen deprivation and castration, it may be possible to cure the disease, or prolong survival significantly.

## Prostate Cancer (PCa)

Cancer is a leading cause of death worldwide, with more than 8.2 million deaths in 2012, and PCa is the leading cause of cancer-related deaths among males^1^. When localized, PCa may be cured by surgery; however, once PCa has become metastatic, androgen-deprivation therapy (ADT) is the mainstay first-line therapy with clinical improvements in more than 90% of patients. However, ADT is not curative; cancer control and palliation only lasts for about 18−24 months until the tumor becomes castration resistant (CRPC) (1).

Until recently, few treatment options were available for metastatic CRPC. However, during recent years, there has been a rapid increase in the number of novel therapies, including the androgen synthesis inhibitor abiraterone, the antiandrogen enzalutamide, the chemotherapeutic taxane cabazitaxel, immunotherapeutic sipuleucel-T, and ^223^Ra-dichloride (Xofigo^®^) targeting active bone cells due to its similarity to calcium (2, 3). ^223^Ra-dichloride is sometimes referred to as a targeted alpha-particle therapy (TAT), although the concept most often is used when alpha particles are directly targeted to the malignant cells in question. Despite recent survival improvements for men with metastatic CRPC, this disease stage remains incurable. Understanding the biology behind drug resistance development and the CRPC stage is of crucial importance if we are to identify and develop new treatment strategies as well as better prognostic and predictive biomarkers for this patient group. Current challenges include both monitoring when CRPC develops and to develop novel therapies that could treat this fatal stage. CRPC is usually suspected in patients with a rising prostate-specific antigen level, or with new evidence of disease on a ^99m^Tc-medronic acid-based bone scintigraphy scan. ^99m^Tc-medronic acid is a phosphate derivate that can replace bone phosphate in areas with reactive bone tissue due to metastases, and bone scintigraphy is the standard procedure for the detection of bone metastases. However, targeted radionuclide pharmaceuticals, for either diagnosis or therapy, may instead specifically target malignant metastatic cells, and regardless of localization (bone or soft tissue), more accurately stage or treat metastatic lesions and better detect relapsed disease, monitor development of CRPC, and predict the aggressiveness of the disease. Novel radiopharmaceuticals may also be developed and used for theranostic purposes, i.e., combined imaging (or multimodal imaging) and therapy utilizing, for example, single-photon emission computed tomography, positron emission tomography, magnetic resonance imaging, ultrasound imaging, Cherenkov luminescence imaging, or photoacoustic imaging, in conjunction with a radioimmunotherapy (RIT)-based (using, for example, beta- or alpha-particle emitters) treatment.

To date, the only prognostic PCa biomarker approved by the US Food and Drug Administration is circulation tumor cell counts (CTCs) (4). One may envision that genomic or proteomic profiling of relapsing CTCs can provide novel prognostic or predictive biomarkers to aid in the development of whole-organ and body imaging and therapy of CRPC patients.

## Resistance and Cancer Stem Cells (CSCs)

The development of CRPC and resistance as well as metastatic spread likely involves reactivation/activation of the cells that initiate metastasis, the CSCs. These are cells that are thought to have the ability to spread to and infiltrate other organs where they can establish dormant or fully fledged tumors, survive, self-renew, and interact with the niche to support daughter cell proliferation and differentiation to recapitulate the cell types of the original tumor (5). Thus, the CSC concept provides a model for the phenotypic and functional heterogeneity found in PCa tumors (6). The model may also explain why patients are not cured when treated with standard therapies—small subpopulations of cancer cells survive because they do not at all, or not to any substantial degree, express the biomarker or pathway of the more differentiated cells that are targeted. Alternatively, the targeted pathways are wired differently in the CSC population. Further, CSCs are believed to show increased radio- and chemoresistance due to increased expression of antiapoptotic proteins, DNA repair genes, and transporter proteins such as ATP-binding cassette (ABC) proteins and aldehyde dehydrogenase (ALDH) genes (7).

On a molecular level, it is clear that progression to CRPC is characterized by an active androgen pathway, despite systemic androgen deprivation, and despite the new repertoire of therapeutic options available today, there is an increase in intratumoral androgen biosynthesis, aberrant androgen receptor (AR) expression, cross talk with other oncogenic pathways, reactivation of epithelial–mesenchymal transition (EMT) processes, and upregulation of genes that regulate stemness and self-renewal (8). The co-expression of AR and several stem cell (SC) markers in CRPC suggest that the AR is aberrantly reactivated together with deregulation of epigenetic mechanisms controlling self-renewal or lineage commitment of SCs. The AR recruits corepressors and histone deacetylases (HDACs) to promoters and thereby re-organizes the chromatin structure to epigenetically modulate transcription of genes that control proliferation and differentiation (9).

Whether CSCs arise from mutated adult tissue SCs, or if CSCs arise from differentiated/lineage committed progenitor cells by means of mutation or epigenetic change to function as a cancer-initiating cell in prostate carcinogenesis and progression is not known. By mutation of pathways that control self-renewal, differentiation may lead to increased stemness. It has, for example, been suggested that induced pluripotent stem cell technology could occur naturally by means of mutation or upregulation of genes that induce or maintain plasticity to reprogram cells into a pluripotent state (10). The EMT process is not only involved in embryogenesis but also involved in tissue repair. During the EMT process, cells acquire mesenchymal properties and it has been reported that EMT is mechanistically linked with SC signatures in PCa cells (11). However, no standardized CSC markers have been established for PCa, or other solid tumors, and different researchers use different surface biomarkers or assays for the investigation of putative CSC biology. Better identification and characterization of normal SCs and CSCs may allow screening of CSC genes or cellular products, including cell surface biomarkers, transcription factors, microRNAs, and exosomes.

## Targeted Alpha-Particle RIT

The most successful clinical application of RIT has until today been achieved by using the beta-particle emitting radionuclides ^90^Y or ^131^I labeled to monoclonal antibodies (mAbs) in the treatment of CD20 expressing follicular B-cell non-Hodgkin’s lymphoma (12). However, regarding RIT treatment of solid cancers, e.g., as adjuvant therapy after primary surgery and/or chemotherapy, there has been limited success. And so far, there is only one FDA approved radionuclide-based drug against PCa as mentioned previously; ^223^Ra-dichloride (Xofigo^®^), although not a RIT approach since the alpha-particle emitter ^223^Ra is not labeled to any mAb.

Targeted alpha therapy (TAT), in which an alpha-particle emitting radionuclide is labeled to a molecular carrier targeting a specific tumor antigen, differs from standard radioimmunotherapeutic approaches emitting beta particles. The quantum mechanical tunneling phenomena enabling the alpha-particle decay results in the release of a highly energetic and heavy alpha particle, consisting of two protons and two neutrons, from the atomic nucleus. If released in tissue, an alpha particle deposit its entire kinetic energy along a 60−90 μm short track, i.e., in the range of only three to six cell diameters. This short range makes alpha particles especially well suited for targeting small tumor cell clusters, or even single tumor cells, and when trying to minimize irradiating non-targeted surrounding healthy tissue. The high frequency of energy deposits within such a short range means a high linear energy transfer (LET), expressed as kiloelectronvolts per micrometer (keV/μm). The five alpha-particle emitters most often used in TAT so far, and that would be suitable for TAT against CSCs, are Actinium-225 (^225^Ac), Radium-223 (^223^Ra), Bismuth-213 (^213^Bi), Bismuth-212 (^212^Bi), and Astatine-211 (^211^At). Actinium-225 emit four alpha particles in a serial decay and have good therapeutic potential, especially if radiochemistry can produce stable binding of ^225^Ac and its daughters to the carrier molecule and/or the ^225^Ac radioimmuno-complex can be rapidly internalized. Radium-223 emits four alpha particles and two beta particles in its decay chain and has been used in the form of ^223^Ra-dichloride against skeletal PCa metastases as mentioned above. This nuclide can be produced via neutron activation of ^226^Ra. Bismuth-213 emits one alpha particle and can be obtained by the elution of a ^225^Ac/^213^Bi generator produced by, for example, the Institute for Transuranium Elements in Karlsruhe, Germany (13). Bismuth-212 emits one alpha particle and one beta particle during its decay. A high-energy gamma ray of 2.6 MeV is accompanying the alpha and beta decay via the daughter nuclide ^208^Tl, and therefore, patients must be kept under radiation protection conditions if this nuclide is to be utilized. If a proper carrier is chelated with its mother nuclide ^212^Pb, this could be used as a vehicle to carry the alpha-particle emitter ^212^Bi to its targets. Astatine-211 emits one alpha particle during either one of its two possible decay routes to stable ^207^Pb. One of this nuclide’s favorable characteristics is that its chemistry is similar to that of Iodine. Due to the fact that this alpha-particle emitter is cyclotron produced, this radionuclide, otherwise highly suitable for TAT, has not so far reached its full potential, as only a handful of research centers and hospitals have this capability. Table 1 presents the abovementioned suitable alpha-particle emitters for TAT, together with their most important physical characteristics. Table 1 also includes the lists the alpha-particle emitter ^227^Th, which also have properties making it a potential TAT nuclide.

**Table 1:** **Alpha-particle emitting radionuclides possible to use for TAT against cancer stem cells (CSCs)**.

Radionuclide	Energy^a^ (keV)	Range^b^ (μm)	LET^c^ (keV/μm)	Half-life^d^
Astatine-211 (^211^At)^e^	7,450 (6,786)	66 (57)	71−240 (125)	7.2 h
Bismuth-212 (^212^Bi)^f^	8,785 (7,804)	86 (71)	63−240 (120)	60.6 min
Bismuth-213 (^213^Bi)^g^	8,377 (8,325)	80 (79)	65−240 (120)	45.6 min
Radium-223 (^223^Ra)^h^	7,386 (6,595)	65 (54)	71−240 (125)	11.4 d
Actinium-225 (^225^Ac)^i^	8,377 (6,877)	80 (58)	65−240 (120)	10.0 d
Thorium-227 (^227^Th)^j^	7,386 (6,458)	65 (53)	71−240 (125)	18.7 d

*Alpha-particle energy, range and linear energy transfer (LET) in soft tissue, and physical half-life are stated*.

*^a^Most abundant alpha-particle energy. Approximate average energy in parenthesis. During the ^225^Ac decay chain, a 7067 MeV particle is approximately as abundant as the 8377 MeV particle stated*.

*^b^Ranges in soft tissue (e.g., skeletal muscle and prostate; 1.04 g/cm^3^) of most abundant-energy (and average-energy) alpha particles. Calculated with James Ziegler’s SRIM-2013 software*.

*^c^Interval of most abundant alpha particle, from initial value to Bragg peak. Approximate average LET in parenthesis. Calculated using James Ziegler’s SRIM-2013 software*.

*^d^Abbreviations: d, days; h, hours; min, minutes*.

*^e^Both decay routs from ^211^At to ^211^Po and ^207^Bi taken into account*.

*^f^Both decay routs from ^212^Bi to ^212^Po and ^208^Tl taken into account*.

*^g^Both decay routs from ^213^Bi to ^213^Po and ^209^Tl taken into account*.

*^h^Decay chain from ^223^Ra to ^211^Po and ^207^Tl taken into account*.

*^i^Decay chain from ^225^Ac to ^213^Po and ^209^Tl taken into account*.

*^j^Decay chain from ^227^Th to ^213^Po and ^209^Tl taken into account*.

High-LET radiation, such as alpha particles (LET ≈ 100 keV/µm), causes damage to the DNA, e.g., frequent double-strand breaks, which are much more difficult to repair by the cell than those most commonly caused by low-LET radiation such as beta-particles (LET ≈ 0.2−0.3 keV/μm), e.g., base damage or single-strand breaks. This high-LET characteristic of alpha-particles makes them independent of oxygenation or active cell proliferation in order to sterilize a cell, and very few hits to a cell are needed in order to sterilize it compared to beta particles. Even one single hit by an alpha particle has a significant chance of sterilizing a cell, although most often approximately 20 hits are considered to be lethal, compared to approximately 2,000 beta particles (14). This type of highly cytotoxic type of radiation, directed against specific tumor cell antigens, has therefore good chance of substantially adding to hitherto failing curative adjuvant treatments for different types of cancer, e.g., prostate, breast, colon, and ovarian cancer, and is sometimes referred to as *systemic conformal radiotherapy at the cellular level*. Several preclinical studies have been performed using TAT, but until today only a limited number of early-stage clinical studies have been completed, as mentioned below.

The TAT methodology against recurrent brain tumors have been used with the alpha-particle emitters ^213^Bi and ^211^At (15, 16). Intraperitoneal TAT in ovarian cancer patients has been performed using ^211^At (17), as well as several preclinical studies evaluating this methodology (18–20). Pharmacokinetics and imaging in patients using ^212^Pb-TCMC-Trastuzumab, directed against human epidermal growth factor receptor-2-positive intraperitoneal ovarian cancer has also been performed (21). A number of studies have been performed using ^213^Bi or ^225^Ac for the treatment of myelogenous leukemia (22–26). TAT of B-lineage non-Hodgkin’s lymphoma using ^213^Bi-labeled anti-CD19- and anti-CD20-CHX-A″-DTPA conjugates has also been performed in one study (27). Against metastatic melanoma ^213^Bi has been used in two studies (28, 29). And as mentioned above, use of ^223^Ra-dichloride is now approved by the FDA against symptomatic skeletal PCa metastases (3).

Regarding the dosimetry of alpha particles, it could under some circumstances be challenging, especially considering the alpha particle’s short path length and high LET in tissue. When the statistical variation of the energy imparted to different cell nuclei caused by alpha particles is high, the average absorbed dose could be of little or no value and microdosimetry might instead be necessary to apply. Kellerer and Chmelevsky suggested already in a paper from 1975 that the stochastic variation of the deposited energy within a specified target, i.e., microdosimetry, must be taken into consideration in situations during which the deviation of a local absorbed dose exceeds 20% (30). MIRD Pamphlet No. 22 gives a comprehensive presentation of alpha-particle radiobiology and dosimetry, and other both review and original papers present different aspects of the subject (14, 31–34).

## Targeted Alpha Therapy Against Prostate CSCs

**Figure 1:**
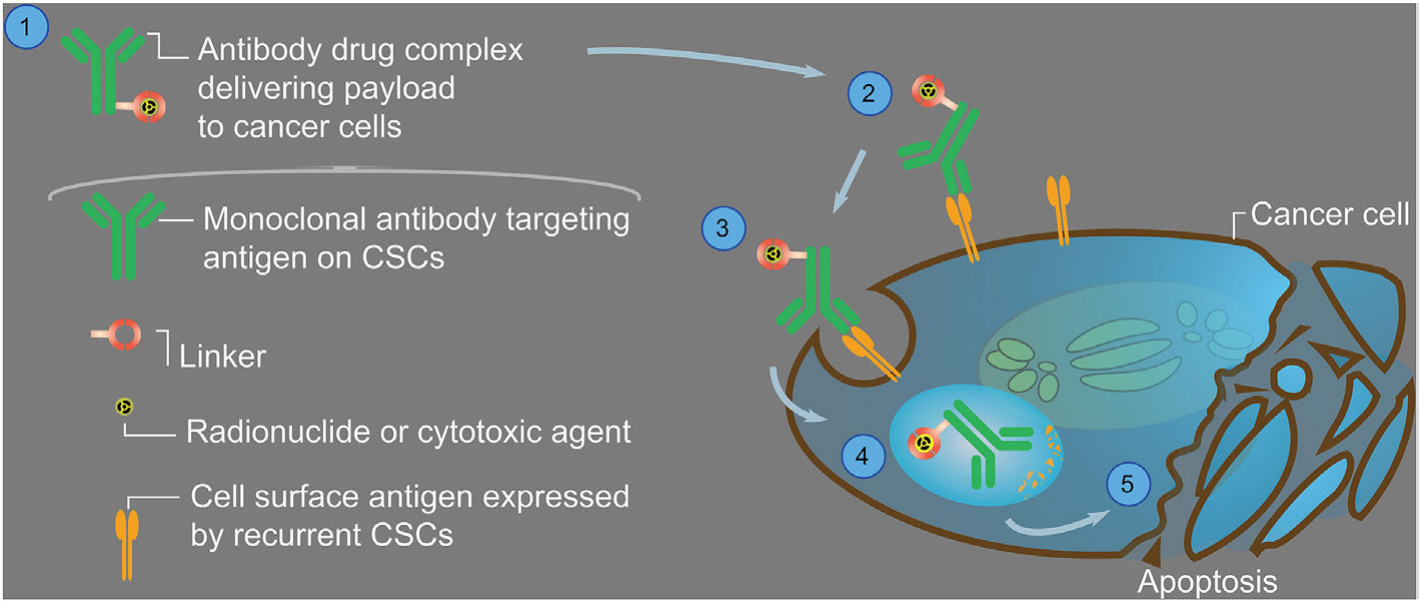
**Generalized image showing an antibody–drug complex (1), and a schematic way in which it is targeted to a cancer stem cell (CSC)**. The antibody should be specific for CSC-associated antigens so that its payload, in the shape of a therapeutic agent, is not delivered to normal cells causing unwanted side effects. The linker should firmly attach the therapeutic agent to the antibody, enabling delivery of the drug to its target (2), and not being released during circulation. The therapeutic agent should be able to kill the targeted CSC when the antibody–drug complex is either bound to the cell surface or internalized in sufficient numbers (3). Receptor-mediated endocytosis (4) of the payload (e.g., an alpha-particle emitting radionuclide or a cytotoxic agent) may cause disruption of cellular processes (including microtubule networks), mitotic arrest, or cell death due to double-strand breaks of the DNA (5). If the targeted antigen on all of the CSCs is sufficiently expressed, the therapeutic agent may eradicate the relapse-causing cells and eventually stop the disease. If for example beta-particles emitted from ^90^Y or ^177^Lu (maximum energy, maximum range, and average linear energy transfer (LET) equal to 2.3 and 0.5 MeV, 11.3 and 1.8 mm, and 0.18 and 0.20 keV/µm, respectively) are used as therapeutic agents, the number of hits to the CSC required to kill it would be at least in the order of 2,000. However, if alpha particles emitted from for example ^211^At or ^213^Bi (maximum energy, maximum range, and average LET equal to 7.4 and 8.4 MeV, 66 and 80 µm, and 125 and 120 keV/µm, respectively) are used instead, the number of hits required would instead be in the order of 20. This means that the likelihood of eradicating the CSCs using alpha-particles greatly exceeds that of using beta particles, especially if the targeted antigen is expressed in low numbers on the CSCs. It should also be noted that, although the range of the alpha particles is in the order of typically only two to three cell diameters, some crossfire will still occur of neighboring eventually untargeted cells.

It is clear from the previous discussion that we cannot allow the CSC population to remain untargeted while treating the bulk of the tumor, as is currently done using conventional therapies, if we are to eradicate the entire tumor burden and prevent relapse. However, given that CSCs are more resistant to chemo- and radiotherapies, smart design strategies may not only target the CSC population but do so more efficiently. By utilizing the TAT technology against CSCs, the effect should be more efficient than the present use of ^223^Ra-dichloride, by reaching and targeting the malignant cells directly, and specifically the cells responsible for tumor recurrence. Treating the bulk of the tumor mass with RIT directed against antigens expressed only on the more differentiated cells, and not the CSCs, will probably not deliver a high enough absorbed dose to the radio-resistant CSCs. By bringing the radionuclide in direct contact with the recurrent CSC, the higher expression of antiapoptotic, DNA repair, drug efflux, and detoxifying proteins in CSCs that enhanced their survival (35) may be overcome if alpha particles are utilized (Figure 1). Therefore, supposedly using a cocktail treatment with for example a beta-particle emitter such as ^90^Y directed against the bulk of the differentiated tumor cells, creating a relatively homogenous absorbed dose distribution within the entire tumor (34), plus an alpha-particle emitter such as ^211^At or ^213^Bi directed against the CSCs, could create a high-absorbed dose region, especially were the CSCs are situated, and be highly effective in conventionally resistant tumors.

Cell surface targeting of the CSCs may occur via monospecific antibodies against various epitopes expressed by CSCs, e.g., CD44, CD133, CD151, or via bispecific antibodies that recognizes two different epitopes. Dual specificity opens up the possibility for enhancing specificity and/or dual blocking of different pathways simultaneously, including directing immune cells to the tumor cells. It may also be used to deliver nanoparticles (NPs) at a high concentration and release them within the tumor cell following uptake by CSCs, e.g., targeting transcription factors or proteins that control DNA transcription such as NF-κB (nuclear factor kappa-light-chain-enhancer of activated B cells), disrupting its pathway and providing opportunity to eradicate the CSC population. Likewise, liposome NPs may be utilized together with alpha-particle emitters to simultaneously deliver chemotherapeutic drugs, most probably enhancing the therapeutic efficacy.

Epigenetic changes may further induce differentiation and reduce SC characteristics in treated cells, including radioresistance (unpublished data). Epigenetic modifications may turn on or off critical genes, and we have previously shown that the epigenetic regulator REST is downregulated in CRPC and associated with dedifferentiation and poor prognosis (36). However, treatment with HDAC inhibitors in combination with RIT (such as TAT) may allow a more efficient treatment by epigenetically regulating expression of SC proteins such as ALDH enzymes and ABC transporters (37, 38), potentiating the effects of the alpha particles in the eradication of the CSC population.

## Conclusion

This article claims that choosing TAT against CSCs might be a way forward to increase the probability of prolonged survival, or even cure, for patients having a metastatic cancerous disease. Castration-resistant PCa is today an incurable disease, with only a relatively short mean survival after progression, despite development of new drugs during recent years targeting the bulk of the prostate tumor cells. Targeting the root cause of tumor recurrence with efficient RIT methods would most likely result in prolonged survival compared to the conventional treatment concepts of today. We think that novel alpha particle-based RIT therapies targeting various prostate CSC markers could transform the treatment of resistant metastatic PCa in the near future.

## Author Contributions

Both authors have made substantial, direct, and intellectual contribution to the work and approved it for publication.

## Conflict of Interest Statement

The authors declare that the research was conducted in the absence of any commercial or financial relationships that could be construed as a potential conflict of interest.

## Footnotes

^1^Globocan. Available from: http://globocan.iarc.fr.
